# Whole-Body Dynamics for Humanoid Robot Fall Protection Trajectory Generation with Wall Support

**DOI:** 10.3390/biomimetics9040245

**Published:** 2024-04-19

**Authors:** Weilong Zuo, Junyao Gao, Jiongnan Liu, Taiping Wu, Xilong Xin

**Affiliations:** 1 School of Mechatronical Engineering, Beijing Institute of Technology, Beijing 100081, China; 2 Beijing Advanced Innovation Center for Intelligent Robotics and Systems, Beijing Institute of Technology, Beijing 100081, China

**Keywords:** fall, humanoid robots, wall support, model predictive control, whole-body control

## Abstract

When humanoid robots work in human environments, they are prone to falling. However, when there are objects around that can be utilized, humanoid robots can leverage them to achieve balance. To address this issue, this paper established the state equation of a robot using a variable height-inverted pendulum model and implemented online trajectory optimization using model predictive control. For the arms’ optimal joint angles during movement, this paper took the distributed polygon method to calculate the arm postures. To ensure that the robot reached the target position smoothly and rapidly during its motion, this paper adopts a whole-body motion control approach, establishing a cost function for multi-objective constraints on the robot’s movement. These constraints include whole-body dynamics, center of mass constraints, arm’s end effector constraints, friction constraints, and center of pressure constraints. In the simulation, four sets of methods were compared, and the experimental results indicate that compared to free fall motion, adopting the method proposed in this paper reduces the maximum acceleration of the robot when it touches the wall to 69.1 m/s^2^, effectively reducing the impact force upon landing. Finally, in the actual experiment, we positioned the robot 0.85 m away from the wall and applied a forward pushing force. We observed that the robot could stably land on the wall, and the impact force was within the range acceptable to the robot, confirming the practical effectiveness of the proposed method.

## 1. Introduction

In human living environments, many tasks can be completed by humanoid robots, such as serving coffee in restaurants, cooking in kitchens, and delivering parcels outdoors. These jobs require not only dexterous motor skills but also preparation for dealing with contingencies like accidental falls, sudden power depletion, or signal interruptions. Given the possibility of various emergencies arising during robotic operations, this study focuses on the online generation and motion control of fall protection trajectories for humanoid robots in simple environments.

At present, research on fall protection for humanoid robots focuses on three main aspects: Firstly, using hardware structure protection, as seen in articles [1,2,3,4]. Although hardware protection can cushion the impact force generated by falls, the overall motion ability of the robot is weakened due to the addition of protective materials, resulting in significantly reduced joint flexibility and workspace. Secondly, using bionic methods: (1) imitating human fall behavior and actions, as introduced in articles [5,6,7], by imitating Ukemi motion or self-protective motion to generate different trajectories based on varying fall speeds; (2) collecting human kinematic, dynamic, and physiological data through motion capture systems, which are ultimately converted into the angles or moments of the corresponding joints of the robot, as shown in articles [8,9,10]. Thirdly, learning the optimal location of the multiple contact points through reinforcement learning algorithms or trajectory optimization, as introduced in articles [11,12,13,14,15]. Although these practices can reduce the harm caused by falling, they all overlook the fact that humans design robots to work in human environments, where many objects, such as walls, desks, and other fixtures, can be utilized to prevent falls.

Inspired by this, many scholars have begun to consider utilizing external objects to help stabilize robots when they fall, such as bracing against a wall. For example, Vincent Samy introduced a model predictive control (MPC) method with uniform gravity distribution in his paper [16]. Meanwhile, in another paper [17], he also introduced a phased optimization control method to achieve stable landing of the robot in unstructured environments. However, we can only see the simulation videos in his article, while the actual experimental results are not known. The COMAN robot arm from IIT optimized the rotation angles of each joint using maximum stiffness ellipsoids [18], but this method lacks general applicability due to the unique structure of the robot, which incorporates both rigid and flexible joints. Inspired by human behavior, Wang Shihao introduced an online control method in his articles [19,20], but the miniature size of his robot meant the actual effects were not fully demonstrated. Da Cui simulated a robot bracing against a wall after being disturbed in his paper [21], modeling the robot’s ankle joint as a passive joint. While convenient for simulation, this method would be very dangerous in actual robot applications. The latest article [22] proposes a D-reflex method, which uses neural networks to learn and predict potential wall brace positions for the robot arm during a fall. The downside is that the robot would need to spend significant time retraining the model if the environment changes.

**Figure 1:**
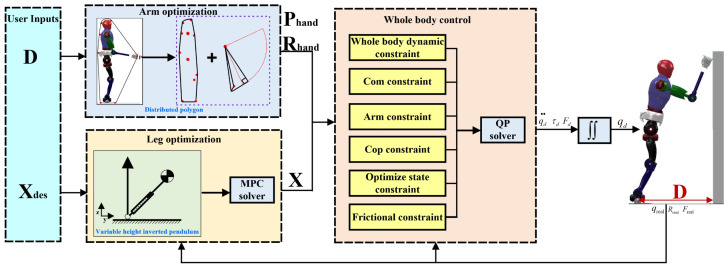
Fall protection trajectory generation for a humanoid robot with wall support. The superscripts “D” denotes the actual distance from the robot to the wall. Other parameters will be introduced in subsequent sections.

To address the problem of utilizing wall to stabilize robots during the process of falling, this study employed a variable height-inverted pendulum model (VHIP) and model predictive control to optimize the center of mass (COM) posture during robot leg squatting. A distributed polygon (DP) method was utilized to calculate the end effector’s pose for the arms. Considering the complexity of coordinating multiple objectives in the falling process, where the superposition of individual task controllers can lead to over-constrained or under-constrained situations, this paper proposes a unified task space whole-body dynamic control method based on quadratic optimization. This method achieves coordinated and stable control of the aforementioned multiple tasks, ensuring dynamic tracking during falling tasks. The schematic diagram of the overall motion framework is illustrated in Figure 1.

The contribution of this work is two-fold:

- An optimized full-body fall protection trajectory for humanoid robots is generated based on variable height-inverted pendulum model and distributed polygon model.
- To address the issue of challenging coordination of multiple objectives during falling scenarios, a quadratic optimization-based unified task space whole-body dynamic control method is proposed. This method involves the design of a multi-objective task PD controller and a set of constraints, enabling the coordinated and stable control of the aforementioned multiple objectives and dynamic tracking of the falling trajectory.
- Four different fall methods were compared, demonstrating the effectiveness of the proposed method in this paper.

## 2. Methods

**Figure 2:**
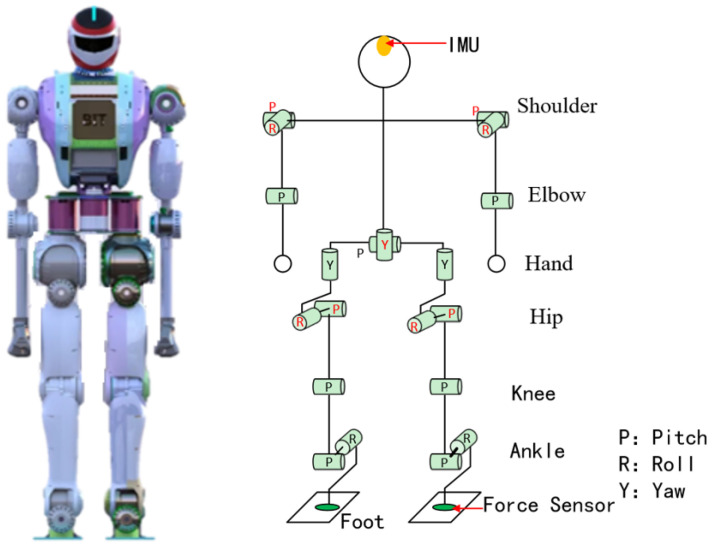
Snapshots of the humanoid robot. The left side represents a three-dimensional view of the robot, and the right side depicts a schematic diagram of the robot’s joints.

The simulation platform used in this study is the FCR humanoid robot, which was independently developed and designed by our laboratory, as shown in Figure 2. The FCR weighs approximately 50 kg, standing about 165 mm tall, each leg has 6 degrees of freedom, the waist has 2 degrees of freedom, the arms each have 4 degrees of freedom, and a total of 22 degrees of freedom. Assuming the optimized joint angle of the robot is *q*, where $q_{b}$ is the value corresponding to the floating base and $q_{j}$ is the angle value of each joint, the following equation can be obtained:

(1) q=qbqj

According to the rigid body dynamics [23], the entire dynamic motion of the robot is shown in Equation (2).

(2) $$H\left(q\right)\ddot{q}+C\left(q,\dot{q}\right)\dot{q}+G\left(q\right)=S\left(q\right)\tau +J_{c}^{T}\left(q\right)F$$

where $H\left(q\right)\in R^{\left(N+6)\times (N+6\right)}$ represents the mass matrix; $C\left(q,\dot{q}\right)\in R^{\left(N+6)\times (N+6\right)}$ represents the matrix of Coulomb forces and $G\left(q\right)\in R^{\left(N+6\right)}$ represents gravity; $S\left(q\right)\in R^{(N+6)\times N}$ represents the joint torque mapping; $\tau \in R^{N}$ represents the joint torque vector; $J_{c}\left(q\right)\in R^{M\times (N+6)}$ is the corresponding contact Jacobian matrix; $F\in R^{M}$ is the M-dimensional external force and torque.

### 2.1. Variable Height-Inverted Pendulum Model

**Figure 3:**
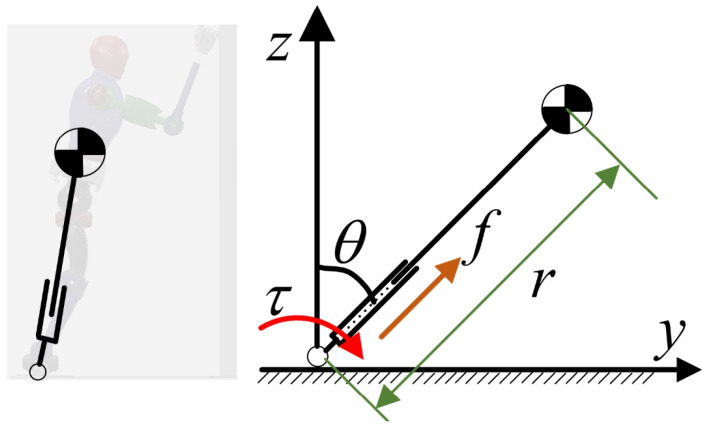
Inverted pendulum model.

During the robot’s fall and support process, we aim to control the falling motion in real time. Compared to traditional full-body dynamics models, using a simplified model not only speeds up computation and reduces the number of calculations, but it is also well-suited for long-duration motions. Due to the variation in the height of the center of mass during the process of robot falling, we employed a variable height-inverted pendulum model to obtain the equations of motion, as shown in Figure 3.

(3) $$r^{2}\ddot{\theta }+2r\dot{r}\dot{\theta }-grsin\theta =\tau /M$$

(4) $$\ddot{r}-r{\dot{\theta }}^{2}+gcos\theta =f/M$$

where *r* represents the length of the link, θ represents the angle at which the rod deviates from the vertical direction, *M* represents the total mass of the robot, *f* represents the force acting along the length of the rod, and τ represents the torque applied at the pivot point. Due to the real-time optimization nature of the method proposed in this paper for generating motion trajectories, which demands high computational efficiency, and since both Equations (3) and (4) contain nonlinear terms, it is necessary to linearize them. Here, we employed a simplified approach based on the model’s behavior near the equilibrium position, represented as $\left[\begin{array}{cccc} \theta & \dot{\theta } & \sin\theta & \cos\theta \end{array}\right]\approx \left[\begin{array}{cccc} 0 & 0 & \theta & 1 \end{array}\right]$.

### 2.2. Model Predictive Control

Model predictive control employs rolling optimization to iteratively optimize the control inputs based on current state variables and input variables. The objective is to minimize a cost function, resulting in the optimal control inputs that steer the output of the controlled variables as close to the desired reference trajectory [24,25].

To enable the robot to deal with different impact forces and distances, this study adopted an online trajectory optimization method. First, the state variables of the robot are defined as $X=[r,\theta ,\dot{r},\dot{\theta }]$, the inputs are defined as U=[f,τ]. Based on the equation mentioned earlier, the simplified dynamic equation is shown below:

(5) $$\left[\begin{array}{c} \dot{r}\\ \dot{\theta }\\ \ddot{r}\\ \ddot{\theta } \end{array}\right]=\left[\begin{array}{cccc} 0 & 0 & 1 & 0\\ 0 & 0 & 0 & 1\\ 0 & 0 & 0 & 0\\ 0 & \frac{g}{r} & 0 & 0 \end{array}\right]\left[\begin{array}{c} r\\ \theta \\ \dot{r}\\ \dot{\theta } \end{array}\right]+\left[\begin{array}{cc} 0 & 0\\ 0 & 0\\ \frac{1}{M} & 0\\ 0 & \frac{1}{Mr^{2}} \end{array}\right]\left[\begin{array}{c} f\\ \tau \end{array}\right]+\left[\begin{array}{c} 0\\ 0\\ -g\\ 0 \end{array}\right]$$

**Figure 4:**
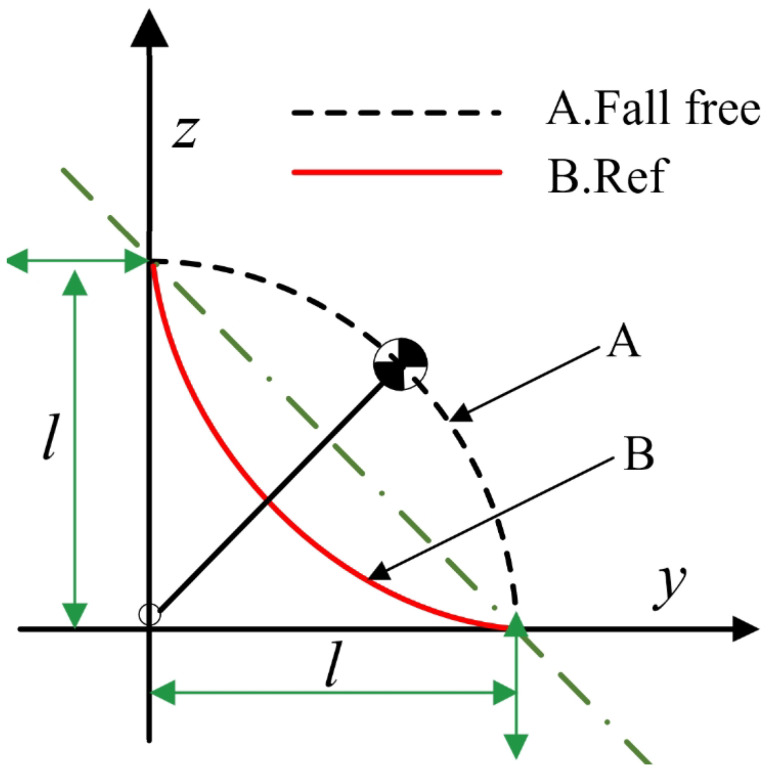
Reference trajectory of the center of mass.

To optimize the state variables and control inputs, we establish the cost function of the robot as shown in Equation (6), where $X^{ref}$ represents the reference values of the state variables. When the link height remains constant and undergoes free fall motion, the trajectory of the center of mass forms an arc, as depicted by curve A in Figure 4. However, considering the variation in the length of the link when the robot falls, we adopted the trajectory represented by curve B. In the absence of considering air friction or energy loss, assuming the robot is in a standing position, the total energy of the system is $E_{1}=mgh+1/2mv^{2}$, where *v* represents the velocity imposed on the robot. As the robot descends, if no protective actions are taken, all the energy will be converted into kinetic energy, resulting in the maximum impact velocity upon landing. If protective actions are taken, a portion of the energy will be converted into potential and kinetic energy, thus reducing the landing velocity. Therefore, during descent, lowering the robot’s center of gravity will decrease the kinetic energy upon landing. This is why we consider adopting the B curve as the reference value.

As for the control input $U^{ref}$, we set it to 0, *Q* and *R* are the corresponding weighting value. Equation (7) represents the discretized form of the state equation, while Equation (8) represents the upper and lower bounds of the control. To effectively solve the aforementioned problem, we transformed Equation (6) into QP (Quadratic Programming) form, as detailed in reference [26]. By reformulating it into QP form, we successfully obtained the state variables *X* and control input *U* of the robot.

(6) $$\min_{X,U}\sum_{i=0}^{k-1}{\left(X_{i+1}-X_{i+1}^{ref}\right)}^{T}Q\left(X_{i+1}-X_{i+1}^{ref}\right)+{\left(U_{i+1}-U_{i+1}^{ref}\right)}^{T}R\left(U_{i+1}-U_{i+1}^{ref}\right)$$

(7) $$s.t.\hat{X}\left[i+1\right]={\hat{A}}_{i}\hat{X}\left(i\right)+{\hat{B}}_{i}{\hat{U}}_{i}$$

(8) $$U_{min}<U_{i}<U_{max}$$

### 2.3. Arm Optimization

**Figure 5:**
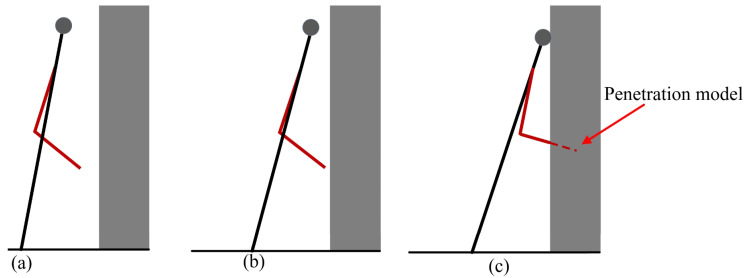
(**a**–**c**) The phenomenon of the robot penetration model.

When a robot falls, the arms play a crucial role. For example, they can be used to adjust the body posture to balance or stabilize the robot’s body. During the falling process, the arms can be utilized to support the robot’s body or to push the robot to alter its motion state, avoiding more severe damage. This article uses the arms to reduce the impact when the robot lands, while also balancing and controlling the robot’s body. Currently, research on robots falling against walls and placing their arms on walls is mainly report in [18,21]. They used the maximum stiffness ellipse method to determine the pose of the robot arm. The problem with this method is that when the robot body has already fallen to a certain position, the arms will undergo a phenomenon of penetrating the model in order to reach the optimized angle value, as shown in Figure 5c. To address this issue, this article uses a multiple contact planning method to reduce the damage to the robot.

When a robot falls, utilizing multiple contact points on its body can better distribute the impact force [27]. This is because by spreading the energy of the robot after landing to multiple contact points, the energy load on a single contact point can be reduced, thereby alleviating the impact force when the robot falls. This method can improve the stability of the robot and reduce the risk of damage to the robot and surrounding environment.

**Figure 6:**
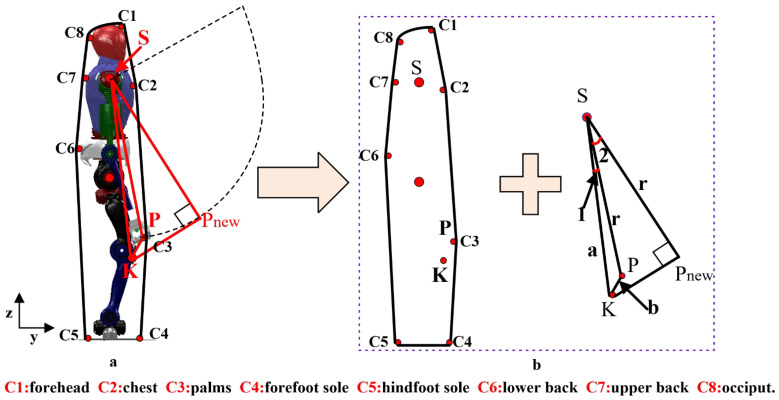
Distributed polygon. (**a**): Represent the points connecting the robot to the ground, (**b**): Represent the corresponding polygon and triangle.

When the robot is standing, connecting these potential contact points on the robot’s body forms a polygon, as shown in Figure 6. These contact points include forehead, chest, palms, forefoot soles, hind-foot soles, lower back, upper back, and occiput, and so on. The length and direction of the sides of this polygon can change. Taking the example of a robot falling forward and reaching out to a wall for support, when the fall was detected, the lower body of the robot begins to squat downward. Compared to other contact points, the knee point *K* was the first to approach the ground. We expect that once the robot’s knee reaches the set reference value, the end of the arm should also touch the wall. Therefore, a trajectory needs to be found for the arm to reach the nearest point. By observing Figure 6a, it can be seen that in the sagittal plane, the workspace of the arm forms a circular area with the upper arm joint as the center and the arm length as the radius. If both the knee and arm need to reach the set reference positions at the same time, the optimal path for the arm is perpendicular to the tangent line passing through the knee point *K*, as shown in $SP_{new}$ in the figure.

In Figure 6a, let the radius of the circle be represented by *r*, and the total length of the arm be denoted by *L*. When the robot arm is fully extended, it not only exert strong impacts on certain joints but also arise singular configurations when solving the inverse kinematics problem. To avoid these issues, this paper introduces a proportionality coefficient, denoted as $k_{1}$, so that $r=k_{1}L$, wherein $0<k_{1}<1$. Based on the previous discussion, point $P_{new}$ represents the intersection of the perpendicular line and the tangent line, and this point’s pose is continuously changing. Initially, the relationship between the upper arm, knee, and arm lengths of the robot is determined. Assuming SK=a, SP=r, PK=b, and ∠KSP=∠1, according to geometric relationships, we can obtain

(9) $$∠1=arccos(a^{2}+r^{2}-b^{2}/2ar)$$

Since $P_{new}S$ is perpendicular to $KP_{new}$, we can conclude that

(10) ∠1+∠2=arccos(r/a)

Therefore, we can determine the rotation angle of the robot’s arm as ∠2. With this angle and the arm length *r*, we can determine the posture of the robot’s arm endpoint. Another point to consider is the distance between the robot and the wall, which also affects the selection of the radius. Let *D* represent the distance between the robot and the wall, and let $r_{1}$ represent the minimum value between PS and KS. When *D* is less than $r_{1}$, we choose the radius of the circle as *D*, else we choose the radius of the circle as $r_{1}$, next we provide pseudo code for this selection process, as shown Algorithm 1.
**Algorithm 1** Calculate the posture of the end point of the arm $P_{\mathrm{new}}$ get KS PS KP *D S* value
 $r_{1}$ = min ($k_{1}PS$, KS)
 **if** 
D< 
$r_{1}$ **then**
  r=D
 **else**
  *r* = min ($k_{1}PS$, KS)
 **end if**
 $\theta_{1}=∠\left(PS,KS\right)$
 $\theta_{2}=∠\left(PS,P_{\mathrm{new}}S\right)$
 **return** 
$P_{\mathrm{new}}=S+\mathrm{RotX}\left(\theta_{2}\right)r$


## 3. Whole-Body Controller

Through the aforementioned variable height-inverted pendulum model and model predictive control method, we obtained the state variables of the robot. Using the distributed polygon method, we obtained the pose of the robot’s arm. Through traditional inverse kinematic methods we can obtain the joints angle, and this method cannot maintain balance in multi-contact situations. Based on this, this paper adopts a whole-body control approach to track the aforementioned reference trajectory. Using whole-body control not only allows the robot to perform multiple tasks simultaneously but also enables coordination between different tasks, thus increasing the robot’s flexibility. Additionally, by controlling multiple joints, whole-body control can enhance the stability of the robot, enabling it to maintain balance in a complex environment. Therefore, this paper adopts a dynamic-based whole-body control approach to track the desired trajectories.

To reduce the impact force upon landing, the robot not only needs to squat its lower body but also requires the arm to touch the wall. This constitutes a multi-objective task. In fulfilling these tasks, the robot also needs to satisfy constraints such as whole-body dynamic constraints, ZMP constraints, friction constraints, and joint limit constraints. To solve for the optimal objective values, we need to set the optimization state variables χ. In this paper, the optimization variables are set as $\chi ={\left[\ddot{q},\tau ,F\right]}^{T}$, where $\ddot{q}$ represents the generalized joint acceleration of the robot, τ is the driving torque, and *F* represents the contact forces and torques at the contact points.

### Constraints and Cost Function

According to the rigid-body dynamics model introduced in Section 2, in order for the robot to reach the target point stably, constraint Equation (2) needs to be satisfied. For the convenience of subsequent optimization, we have modified it as follows:

(11) $$\underset{A_{M}}{\underset{︸}{\left[\begin{array}{ccc} H(q) & -S(q) & -J^{T} \end{array}\right]}}\underset{\chi }{\underset{︸}{\left[\begin{array}{c} \ddot{q}\\ \tau \\ F \end{array}\right]}}=\underset{b_{M}}{\underset{︸}{-C\left(q,\dot{q}\right)\dot{q}}}$$

Let

(12) $$A_{M}=\left[\begin{array}{ccc} H(q) & -S(q) & -J^{T} \end{array}\right]$$

(13) $$b_{M}=\left[\begin{array}{c} -C\left(q,\dot{q}\right)\dot{q} \end{array}\right]$$

During the slow walking process of the robot, it is common practice to place the center of mass of the robot between the two hip joints. This simplified approach is suitable for slow walking speeds. However, during more dynamic motions such as falling, the robot’s actions can be relatively intense. Therefore, we determine the actual position of the center of mass based on the real positions, orientations, and masses of the robot’s links. The actual position of the robot’s center of mass can be expressed as follows:

(14) $$p_{com}=\frac{\sum_{i=1}^{N}m_{i}p_{i}}{\sum_{i=1}^{N}m_{i}}$$

Similarly, we can obtain the direction of the robot’s center of mass, denoted as $R_{com}$. Let $p_{com}^{d}$ represent the desired center of mass position obtained from Section 2.2, and $R_{com}^{d}$ represent the desired center of mass direction. While in motion, we aim to achieve stable control of the COM pose. Following the approach in [28], we have used a PD feedback control to modify the output of the task space controller, resulting in the following equation:

(15) $${\ddot{p}}_{com}^{*}={\ddot{p}}_{com}^{d}+k_{p}\left(p_{com}^{d}-p_{com}\right)+k_{d}\left({\dot{p}}_{com}-{\dot{p}}_{com}^{d}\right)$$

(16) $${\ddot{R}}_{com}^{*}={\ddot{R}}_{com}^{d}+k_{R}\left(R_{com}^{d}-R_{com}\right)+k_{D}\left({\dot{R}}_{com}-{\dot{R}}_{com}^{d}\right)$$

By taking the second derivative of the task space control objectives $p_{com}$ and $R_{com}$, we can obtain the following equations:

(17) $${\ddot{p}}_{com}=J_{p}^{com}\left(q\right)\ddot{q}+{\dot{J}}_{p}^{com}\left(q\right)\dot{q}$$

(18) $${\ddot{R}}_{com}=J_{R}^{com}\left(q\right)\ddot{q}+{\dot{J}}_{R}^{com}\left(q\right)\dot{q}$$

By combining Equations (15)–(18) and considering that we desire the task space output of the robot to be equal to the actual output, we can obtain the following state equation:

(19) $$\underset{A_{c}}{\underset{︸}{\left[\begin{array}{ccc} J_{p}^{com}\left(q\right) & 0 & 0\\ J_{R}^{com}\left(q\right) & 0 & 0 \end{array}\right]}}\underset{\chi }{\underset{︸}{\left[\begin{array}{c} \ddot{q}\\ \tau \\ F \end{array}\right]}}=\underset{b_{c}}{\underset{︸}{\left[\begin{array}{c} -{\dot{J}}_{p}^{com}\left(q\right)\dot{q}+{\ddot{p}}_{com}^{*}\\ -{\dot{J}}_{R}^{com}\left(q\right)\dot{q}+{\ddot{R}}_{com}^{*} \end{array}\right]}}$$

Similarly, we can derive the control equation for the end effector trajectory of the arm, denoting it as Equation (20). When the entire body of the robot moves forward, the end position of the robot’s arm must reach the wall, and the distance is *D*.

(20) $$\begin{array}{cc} A_{arm}\chi & =b_{arm}\\ p_{hand} & =D \end{array}$$

The constraints introduced earlier are equality constraints. However, when the robot falls and comes into contact with a wall, we do not want it to experience backward sliding. Therefore, it is necessary to set up relevant friction constraints. In this paper, the friction coefficient is denoted as μ=0.75, and the friction constraint can be represented by the following equation:

(21) $$\begin{array}{cc} \; & -\mu F_{z}\leq F_{x}\leq \mu F_{z}\\ \; & -\mu F_{z}\leq F_{y}\leq \mu F_{z}\\ \; & 0<F_{min}\leq F_{z}\leq F_{max}\\ \; & \tau_{min}\leq F_{x}\leq F_{z} \end{array}$$

Simplifying the above Equation (21), we obtain

(22) $$A_{\mu }\chi \leq b_{\mu }$$

In addition, during the process of falling, the center of pressure (COP) should be within the support polygon of the robot, which corresponds to the contact area of the supporting feet. Therefore, we can derive the corresponding equation as follows:

(23) $$\begin{array}{cc} P_{copx} & =-\frac{\tau_{c,y}-f_{c,x}d_{s}}{f_{c,z}}\\ p_{copy} & =-\frac{\tau_{c,x}-f_{c,y}d_{s}}{f_{c,z}}\\ -\frac{l_{Foot}}{2} & <P_{copx}<\frac{l_{Foot}}{2}\\ -\frac{w_{Foot}}{2} & <p_{copy}<\frac{w_{Foot}}{2} \end{array}$$

where $\tau_{c,x}$ denote the moment generated by the supporting leg of the robot along the x-axis, $f_{c,y}$ is the force along the y-axis, and $f_{c,z}$ is the force along the z-axis. $l_{foot}$ and $w_{foot}$ represent the length and width of the humanoid robot’s footplate, respectively, and $d_{s}$ represents the mounting height of the six-axis force/torque sensor.

Simplifying the above Equation (23), we obtain

(24) $$A_{cop}\chi \leq b_{cop}$$

The optimization state variable of the robot should also be within its corresponding range, as follows:

(25) $$\chi_{min}\leq \chi \leq \chi_{max}$$

By using the above equation, we obtain the constraints for the robot during its motion. However, these constraints can also be transformed into objective functions [29]. One common approach is to convert them into the form of quadratic programming. We define the cost function as a multi-objective optimization function, which is specified as follows. The first term represents that the robot needs to satisfy the whole-body dynamic equation, which needs to be satisfied at all times during the motion process, so its weight coefficient $W_{i}\left(i=1,2,3,4,5\right)$ is set relatively large. The second term represents the constraint on the pose of the center of mass, the third term represents the constraint on the arms pose, the fourth term represents the friction constraint, the fifth term represents the requirement for the robot to stay within the center of pressure range during its motion.

(26) $$\begin{array}{c} \min_{\chi }W_{1}\left(A_{M}\chi -b_{M}\right)+W_{2}\left(A_{c}\chi -b_{c}\right)+W_{3}\left(A_{arm}\chi -b_{arm}\right)+W_{4}\left(A_{\mu }\chi -b_{\mu }\right)+W_{5}\left(A_{cop}\chi -b_{cop}\right) \end{array}$$

(27) $$s.t.A_{M}\chi =b_{M}$$

(28) $$A_{c}\chi =b_{c}$$

(29) $$A_{arm}\chi =b_{arm}$$

(30) $$p_{\mathrm{hand}}=D$$

(31) $$A_{\mu }\chi \leq b_{\mu }$$

(32) $$A_{\mathrm{cop}}\chi \leq b_{\mathrm{cop}}$$

(33) $$\chi_{min}\leq \chi \leq \chi_{max}$$

## 4. Simulation

To validate the effectiveness of the proposed method, we conducted simulations using MATLAB R2023a and CoppeliaSim 4.1 software. MATLAB was primarily utilized for optimizing equations related to model predictive control and quadratic programming. CoppeliaSim, on the other hand, received joint position commands from MATLAB and provided MATLAB with actual joint positions, floating base orientations, and contact forces. Kinematics and dynamics calculations for the robot were performed using the Frost software [30]. The simulation was conducted within the Bullet 2.78 dynamics engine with a control cycle of 5 ms. In the model predictive control, the prediction horizon was set to 10, and the weight values for matrix *Q* were chosen as [1000,1000,100,40], while the weight values for matrix R were set as [1e−3,1e−3]. The values for $X_{ref}$ were determined according to Section 2.2, and all $U_{ref}$ values were set to 0.

At the beginning, we applied a forward force of 200 N and a last time of 0.2 s on the robot’s head. The detection of whether the robot would fall and the direction of the fall were determined based on reference [31]. The distance *D* between the robot’s ankle and the wall was provided in advance. In the simulation, the distance is 1 m. To validate the practical effectiveness of the proposed method, this study conducted several comparative simulations. The first simulation group involved the robot without any protective actions. In the second group, the robot generated leg joint trajectories solely using the variable height-inverted pendulum model. In the third group, the combination of the variable height-inverted pendulum model and the distributed polygon method was employed. Finally, the fourth group expanded upon the third group by incorporating whole-body motion control.

### 4.1. Falling without Protective Actions

**Figure 7:**
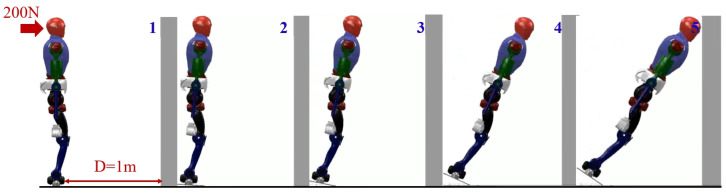
Humanoid robot without taking any protective actions.

**Figure 8:**
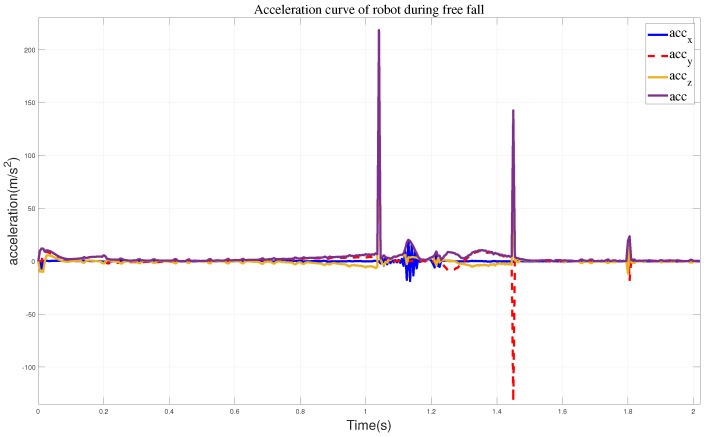
The curves of a robot performing free fall motion, where acc represents all sum accelerations.

In the first set of simulation experiments, when the robot did not take any protective actions, it directly toppled into the wall. Figure 7 illustrates the robot’s motion state, while Figure 8 represents the corresponding impact acceleration. Based on this, it can be observed that the maximum acceleration is 218.45 m/s^2^, and at 1.45 s, the robot collided with the wall again.

### 4.2. Falling with the Variable Height-Inverted Pendulum Model

**Figure 9:**
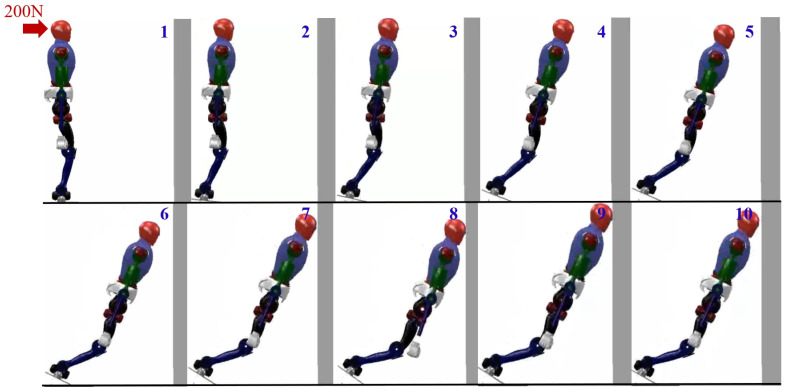
Humanoid robot falling utilizing the variable height-inverted pendulum model.

By employing a variable height-inverted pendulum model and model predictive control, we obtained the state variables *X* of the robot. Using inverse kinematics, we acquired the joint angles of the robot’s legs. The resulting schematic diagram of the robot’s motion is shown in Figure 9. From this, we can observe that without arm support, the robot lowers its center of mass to reduce the impact velocity. As a result, the soles of its feet do not fully contact the ground, indicating a potential tendency for the body to slide downward.

**Figure 10:**
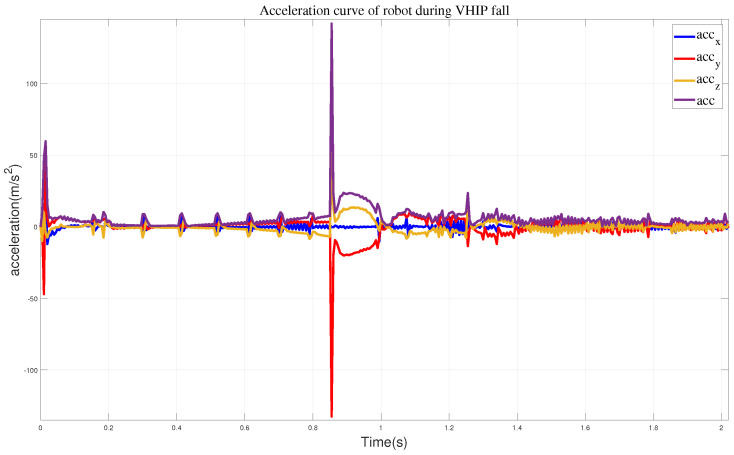
The acceleration curves when employing the VHIP method.

We obtained the impact acceleration when the robot is descending, as shown in Figure 10. Analyzing the latter part, it can be observed that after the robot’s head reaches the wall, a secondary collision occurs between the body and the wall, with a relatively smaller collision velocity, and the maximum acceleration is 141.96 m/s^2^.

### 4.3. Falling with the Variable Height-Inverted Pendulum Model and Distributed Polygon Method

**Figure 11:**
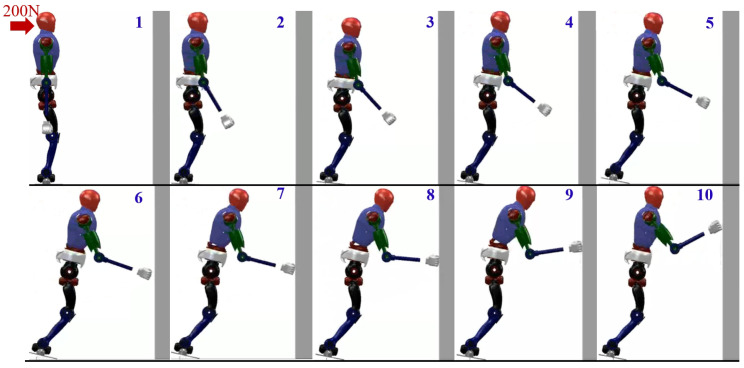
Humanoid robot falling utilizing the variable height-inverted pendulum model and distributed polygon method.

Simulation 2 presents the motion trajectory of the lower body of the robot when utilizing the variable height-inverted pendulum model. From the above figure, it can be observed that the robot almost collapses on the wall during the descent. However, the main focus of this study is the magnitude of the acceleration and balance effect when the robot lands on the wall. In this section, motion control of the arms has been incorporated, and the final result is shown in Figure 11. We can observe that during the robot’s motion, the heel exhibits a phenomenon of being off the ground. Additionally, after the arms make contact with the wall, they undergo a sliding motion along the wall, which in turn affects the movement of the waist joint, as shown in sequences 7, 8, and 9 of Figure 11.

**Figure 12:**
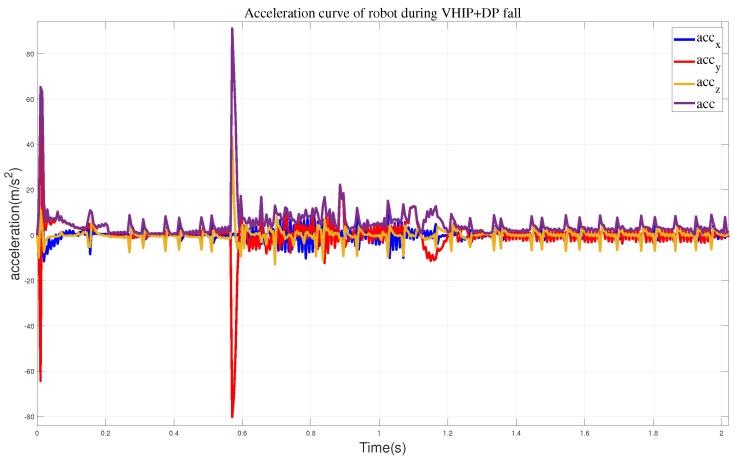
The acceleration curves when employing the VHIP and DP methods.

We also captured the impact acceleration curve of the robot when employing this approach, as depicted in Figure 12. From this, we can observe that the maximum acceleration when the robot reaches the wall is 91.14 m/s^2^, and the maximum acceleration in the y-direction is 80.2 m/s^2^. Compared to the previous two methods, adapting this approach effectively mitigates the impact velocity when the robot lands, but the robot’s soles leave the ground, causing instability. Once an impact occurs, the robot immediately falls down.

### 4.4. Falling with Whole-Body Control

**Figure 13:**
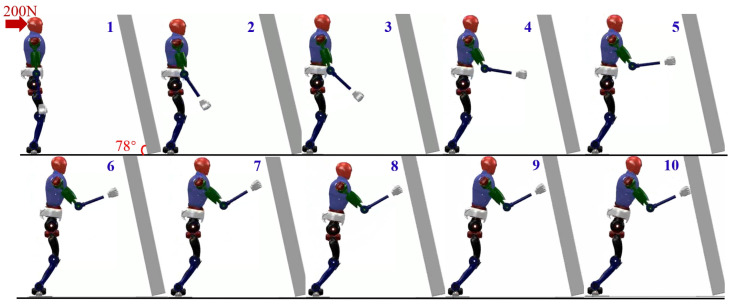
Humanoid robot falling utilizing the variable height-inverted pendulum model, distributed polygon, and WBC method.

In order to validate the adaptability of the proposed variable height-inverted pendulum model, distributed polygon, and whole-body motion control under different environmental conditions, we rotated the wall counterclockwise by 12° and observed the robot’s motion in this state. The final result is shown in Figure 13. It can be observed that during the robot’s motion, the feet maintain continuous contact with the ground. Compared to the method without the added motion control, the robot’s arms smoothly reach the wall without the aforementioned sliding motion causing waist joint deviation.

**Figure 14:**
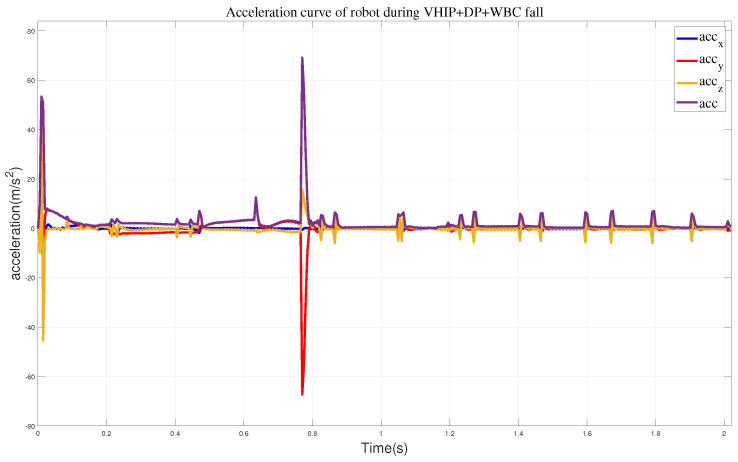
The acceleration curves when employing the VHIP, DP, and WBC methods.

**Figure 15:**
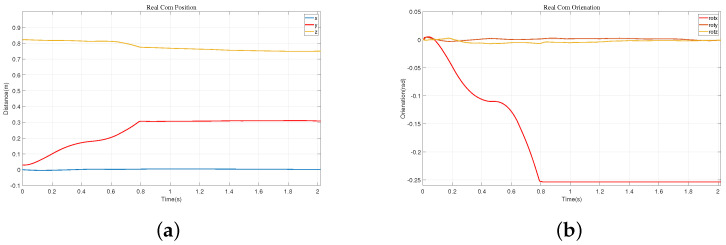
The state variables during the robot falls. (**a**) The position of the center of mass. (**b**) The orientation of the center of mass.

We also obtained the impact acceleration curve of the robot under this method, as shown in Figure 14. It can be seen that at 0.77 s, the robot makes contact with the wall with a maximum acceleration of 69.1 m/s^2^. After reaching the wall, it can maintain a stable state smoothly. Therefore, compared to the previous methods, this approach effectively addresses the impact force when the robot lands. At the same time, we obtained the state variables of the robot during its motion, including the position and direction of the center of mass, as shown in Figure 15. From the state variables, we can observe that the robot’s center of mass descends to 0.75 m, moves forward in the horizontal direction to 0.3 m, and achieves a relatively stable posture after reaching a rotation angle of 14.5°.

## 5. Hardware Experiment

To validate the practical application of the aforementioned methods, we conducted experimental verification on a robot developed exclusively in our laboratory, called FCR-robot. The name FCR stands for Falling, Crawling, and Rolling, and these functionalities are described in detail in references [32,33]. The specific dimensional parameters are listed in Table 1; the robot’s hardware utilizes CAN communication, and the control system employs the RTX real-time operating system with a control cycle of 5 milliseconds. The kinematics and dynamics of the robot are generated using Frost. The prediction horizon and weight coefficients for the MPC are the same as those used in the simulation. To prevent drastic changes in the robot’s joint angles during whole-body control, we implemented limits on the final angle values sent to the robot’s motors, ensuring they do not exceed the predefined threshold.

**Figure 16:**
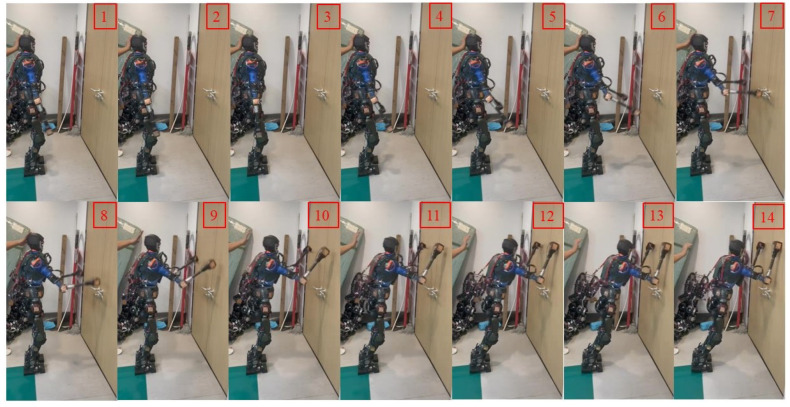
Experiment on the robot’s actual fall and wall support.

**Figure 17:**
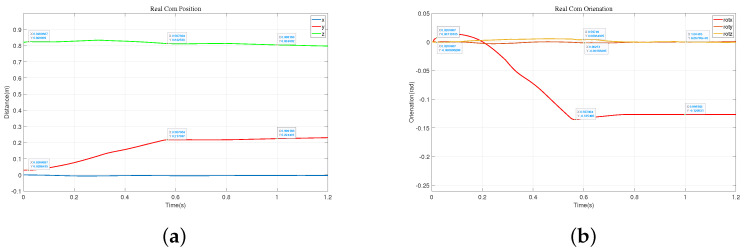
The fall protection motion of the robot when it is 1 m away from the wall. (**a**) The position of the center of mass. (**b**) The orientation of the center of mass.

In the initial stage, the robot maintains a standing posture and is positioned 0.85 m away from the wall. We artificially applied a forward force to the robot, and at this point, the robot detects an imminent fall and immediately swings its arms forward, as shown in (2)–(7) of Figure 16. During the forward swing, the leg joints also rotate forward, as depicted in (8)–(11). Eventually, the robot reaches the wall and maintains a stable posture, as shown in (12)–(14). We obtained schematic representations of the robot’s center of mass position and Euler angles, as shown in Figure 17. Combining these two figures, we can observe that the vertical descent of the robot’s center of mass is about 0.02 m, while the forward displacement is 0.202 m, with a rotation angle of approximately 8.02°.

## 6. Discussion

This study employed a variable height-inverted pendulum model and distributed polygon method to generate the trajectories of the robot’s center of mass and arm. Subsequently, these trajectories were input into a whole-body dynamic control framework for optimization and solution, yielding the acceleration values of each joint. The joint angle trajectories were obtained through second integration. Simulation comparisons were conducted to demonstrate the effectiveness of the proposed method by contrasting it with four different approaches. The results confirmed the superior performance of the method presented in this study. It should be noted that this study focuses on the scenario where the robot stands on both feet and does not consider walking or running. Therefore, future research may address these aspects. Additionally, further investigations will be conducted on employing rolling motions to reduce the impact force during high-impact situations. In conclusion, the proposed method in this study holds significant importance for subsequent research.

## 7. Conclusions

To address the issue of utilizing surrounding objects as support during a robot’s fall, this study first established a variable height-inverted pendulum model and derived model predictive control method to obtain the optimal state variable for the robot. To reduce the impact energy when the robot lands, the arm utilizes a distributed polygon method to reach the nearest point to the wall. To obtain optimal joint trajectories, this study employed whole-body motion control. By assigning different weights to the multi-objective constraints as a cost function and utilizing a QP solver, the joint accelerations were optimized. Constraints such as whole-body dynamics, center of mass, arm configuration, pressure center, and friction were considered. In the simulation, we compared the acceleration curves of free fall, using the variable height-inverted pendulum model, the variable height-inverted pendulum model with the distributed polygon method, and the addition of the whole-body motion control method. The results show that compared to the maximum acceleration of 218.45 m/s^2^ during a free fall, our method can reduce it to 69.1 m/s^2^. Finally, the proposed theory was validated using a physical prototype. By applying a forward force to the robot, it can smoothly reach the target point, confirming the practical effectiveness of the proposed approach.

## 8. Future Work

Since we provided the distance between the robot and the wall in advance in actual experiments, we plan to install SLAM on the robot in future work to achieve real-time online detection.

## Figures and Tables

**Table 1:** The parameters of our robot.

Parameter	Size	Mass
Thigh	361 (mm)	7.36 (kg)
Shank	330 (mm)	5.12 (kg)
Boom	350 (mm)	4.15 (kg)
Jib	360 (mm)	2.3 (kg)
Others	—	31.07 (kg)
Total Mass	—	50 (kg)

## Data Availability

The datasets generated and analyzed during the current study are not publicly available as the data also forms part of an ongoing study but are available from the corresponding author on reasonable request.

## References

[1] Kakiuchi Y., Kamon M., Shimomura N., Yukizaki S., Takasugi N., Nozawa S., Inaba M. Development of life-sized humanoid robot platform with robustness for falling down, long time working and error occurrence. Proceedings of the 2017 IEEE/RSJ International Conference on Intelligent Robots and Systems(IROS).

[2] Kojio Y., Noda S., Sugai F., Kojima K., Kakiuchi Y., Okada K., Inaba M. (2021). Dynamic Fall Recovery Motion Generation on Biped Robot With Shell Protector. IEEE Robot. Autom. Lett..

[3] Kajita S., Cisneros R., Benallegue M., Sakaguchi T., Nakaoka S.I., Morisawa M., Kanehiro F. Impact acceleration of falling humanoid robot with an airbag. Proceedings of the 2016 IEEE-RAS 16th International Conference on Humanoid Robots (Humanoids).

[4] Lee J., Choi W., Kanoulas D., Subburaman R., Caldwell D.G., Tsagarakis N.G. An active compliant impact protection system for humanoids: Application to walk-man hands. Proceedings of the 2016 IEEE-RAS 16th International Conference on Humanoid Robots (Humanoids).

[5] Fujiwara K., Kanehiro F., Kajita S., Kaneko K., Yokoi K., Hirukawa H. UKEMI: Falling motion control to minimize damage to biped humanoid robot. Proceedings of the IEEE/RSJ International Conference on Intelligent Robots and Systems.

[6] Liu D., Lin Y., Kapila V. A rollover strategy for wrist damage reduction in a forward falling humanoid. Proceedings of the 2021 IEEE International Conference on Mechatronics and Automation.

[7] Shimizu T., Saegusa R., Ikemoto S., Ishiguro H., Metta G. Adaptive self-protective motion based on reflex control. Proceedings of the 2011 International Joint Conference on Neural Networks.

[8] Li Q., Chen X., Zhou Y., Yu Z., Zhang W., Huang Q. (2017). A minimized falling damage method for humanoid robots. Int. J. Adv. Robot. Syst..

[9] Meng L., Yu Z., Chen X., Zhang W., Ceccarelli M., Hashimoto K., Liu H. A falling motion control of humanoid robots based on biomechanical evaluation of falling down of humans. Proceedings of the 2015 IEEE-RAS 15th International Conference on Humanoid Robots (Humanoids).

[10] Zhang Z., Huang Q., Liu H., Zhang W., Chen X., Yu Z. Passive buffering arm for a humanoid robot against falling damage. Proceedings of the 2016 IEEE International Conference on Mechatronics and Automation.

[11] Ruiz-del-Solar J., Palma-Amestoy R., Marchant R., Parra-Tsunekawa I., Zegers P. (2009). Learning to fall: Designing low damage fall sequences for humanoid soccer robots. Robot. Auton. Syst..

[12] Luo D., Deng Y., Han X., Wu X. Biped robot falling motion control with human-inspired active compliance. Proceedings of the 2016 IEEE/RSJ International Conference on Intelligent Robots and Systems.

[13] Yun S.K., Goswami A., Sakagami Y. Safe fall: Humanoid robot fall direction change through intelligent stepping and inertia shaping. Proceedings of the 2009 IEEE International Conference on Robotics and Automation.

[14] Wang S., Hauser K. Unified multi-contact fall mitigation planning for humanoids via contact transition tree optimization. Proceedings of the 2018 IEEE-RAS 18th International Conference on Humanoid Robots.

[15] Ha S., Liu C.K. Multiple contact planning for minimizing damage of humanoid falls. Proceedings of the 2015 IEEE/RSJ International Conference on Intelligent Robots and Systems (IROS).

[16] Samy V., Caron S., Bouyarmane K., Kheddar A. Post-impact adaptive compliance for humanoid falls using predictive control of a reduced model. Proceedings of the 2017 IEEE-RAS 17th International Conference on Humanoid Robotics.

[17] Samy V., Bouyarmane K., Kheddar A. QP-based adaptive-gains compliance control in humanoid falls. Proceedings of the 2017 IEEE International Conference on Robotics and Automation (ICRA).

[18] Hoffman E.M., Perrin N., Tsagarakis N.G., Caldwell D.G. Upper limb compliant strategy exploiting external physical constraints for humanoid fall avoidance. Proceedings of the 2013 13th IEEE-RAS International Conference on Humanoid Robots.

[19] Wang S., Hauser K. Real-time stabilization of a falling humanoid robot using hand contact: An optimal control approach. Proceedings of the 2017 IEEE-RAS 17th International Conference on Humanoid Robotics.

[20] Wang S., Hauser K. Realization of a real-time optimal control strategy to stabilize a falling humanoid robot with hand contact. Proceedings of the 2018 IEEE International Conference on Robotics and Automation (ICRA).

[21] Cui D., Peers C., Wang G., Chen Z., Richardson R., Zhou C. (2021). Human inspired fall arrest strategy for humanoid robots based on stiffness ellipsoid optimisation. Bioinspir. Biomim..

[22] Anne T., Dalin E., Bergonzani I., Ivaldi S., Mouret J.B. (2022). First do not fall: Learning to exploit a wall with a damaged humanoid robot. IEEE Robot. Autom. Lett..

[23] Featherstone R. (1984). Robot dynamics algorithms. Annexe Thesis Digitisation Project 2016 Block 5. Ph. D. Thesis.

[24] Murooka M., Morisawa M., Kanehiro F. (2022). Centroidal trajectory generation and stabilization based on preview control for humanoid multi-contact motion. IEEE Robot. Autom. Lett..

[25] Chignoli M., Kim D., Stanger-Jones E., Kim S. The MIT humanoid robot: Design, motion planning, and control for acrobatic behaviors. Proceedings of the 2020 IEEE-RAS 20th International Conference on Humanoid Robots (Humanoids).

[26] Li J., Nguyen Q. Force-and-moment-based model predictive control for achieving highly dynamic locomotion on bipedal robots. Proceedings of the 2021 60th IEEE Conference on Decision and Control (CDC).

[27] Subburaman R., Lee J., Caldwell D.G., Tsagarakis N.G. Online falling-over control of humanoids exploiting energy shaping and distribution methods. Proceedings of the 2018 IEEE International Conference on Robotics and Automation (ICRA).

[28] Wensing P.M., Orin D.E. High-speed humanoid running through control with a 3D-SLIP model. Proceedings of the 2013 IEEE/RSJ International Conference on Intelligent Robots and Systems.

[29] Bertsekas D.P. (2017). Nonlinear Programming.

[30] Hereid A., Ames A.D. FROST: Fast Robot Optimization and Simulation Toolkit. Proceedings of the 2017 IEEE International Conference on Intelligent Robots and Systems (IROS).

[31] Wu T., Yu Z., Chen X., Dong C., Gao Z., Huang Q. (2021). Falling prediction based on machine learning for biped robots. J. Intell. Robot. Syst..

[32] Zuo W., Gao J., Cao J., Xin X., Jin M., Chen X. (2023). Whole-body dynamics-based aerial fall trajectory optimization and landing control for humanoid robot. Biomimetics.

[33] Cao J., Gao J., Zuo W., Liu J., Xin X., Jin M. The Tumbling Motion Planning of Humanoid Robot with Rolling-Stone Dynamics Model. Proceedings of the 2022 IEEE International Conference on Cyborg and Bionic Systems (CBS).

